# Manipulating the Alpha Level Cannot Cure Significance Testing

**DOI:** 10.3389/fpsyg.2018.00699

**Published:** 2018-05-15

**Authors:** David Trafimow, Valentin Amrhein, Corson N. Areshenkoff, Carlos J. Barrera-Causil, Eric J. Beh, Yusuf K. Bilgiç, Roser Bono, Michael T. Bradley, William M. Briggs, Héctor A. Cepeda-Freyre, Sergio E. Chaigneau, Daniel R. Ciocca, Juan C. Correa, Denis Cousineau, Michiel R. de Boer, Subhra S. Dhar, Igor Dolgov, Juana Gómez-Benito, Marian Grendar, James W. Grice, Martin E. Guerrero-Gimenez, Andrés Gutiérrez, Tania B. Huedo-Medina, Klaus Jaffe, Armina Janyan, Ali Karimnezhad, Fränzi Korner-Nievergelt, Koji Kosugi, Martin Lachmair, Rubén D. Ledesma, Roberto Limongi, Marco T. Liuzza, Rosaria Lombardo, Michael J. Marks, Gunther Meinlschmidt, Ladislas Nalborczyk, Hung T. Nguyen, Raydonal Ospina, Jose D. Perezgonzalez, Roland Pfister, Juan J. Rahona, David A. Rodríguez-Medina, Xavier Romão, Susana Ruiz-Fernández, Isabel Suarez, Marion Tegethoff, Mauricio Tejo, Rens van de Schoot, Ivan I. Vankov, Santiago Velasco-Forero, Tonghui Wang, Yuki Yamada, Felipe C. M. Zoppino, Fernando Marmolejo-Ramos

**Affiliations:** ^1^Department of Psychology, New Mexico State University, Las Cruces, NM, United States; ^2^Zoological Institute, University of Basel, Basel, Switzerland; ^3^Swiss Ornithological Institute, Sempach, Switzerland; ^4^Centre for Neuroscience Studies, Queens University, Kingston, ON, Canada; ^5^Faculty of Applied and Exact Sciences, Metropolitan Technological Institute, Medellín, Colombia; ^6^School of Mathematical and Physical Sciences, University of Newcastle, Callaghan, NSW, Australia; ^7^Department of Mathematics, State University of New York at Geneseo, Geneseo, NY, United States; ^8^Quantitative Psychology Unit, Faculty of Psychology, University of Barcelona, Barcelona, Spain; ^9^Institut de Neurociències, University of Barcelona, Barcelona, Spain; ^10^Department of Psychology, Faculty of Arts, University of New Brunswick, Saint John, NB, Canada; ^11^Independent Researcher, New York, NY, United States; ^12^School of Psychology, Benemérita Universidad Autónoma de Puebla, Puebla, Mexico; ^13^Center for Social and Cognitive Neuroscience, School of Psychology, Universidad Adolfo Ibáñez, Santiago, Chile; ^14^Oncology Laboratory, Instituto de Medicina y Biologia Experimental de Cuyo, CCT CONICET Mendoza, Mendoza, Argentina; ^15^School of Statistics, Faculty of Sciences, National University of Colombia, Medellín, Colombia; ^16^School of Psychology, University of Ottawa, Ottawa, ON, Canada; ^17^Department of Health Sciences, Vrije Universiteit Amsterdam and Amsterdam Public Health Research Institute, Amsterdam, Netherlands; ^18^Department of Mathematics and Statistics, Indian Institute of Technology, Kanpur, India; ^19^Biomedical Center Martin, Jessenius Faculty of Medicine, Comenius University, Martin, Slovakia; ^20^Institute of Measurement Science, Slovak Academy of Sciences, Bratislava, Slovakia; ^21^Department of Psychology, Oklahoma State University, Stillwater, OK, United States; ^22^Faculty of Statistics, Saint Thomas University, Bogotá, Colombia; ^23^Department of Allied Health Sciences, College of Health, Agriculture, and Natural Resources, University of Connecticut, Storrs, CT, United States; ^24^Departamento de Biología de Organismos, Universidad Simón Bolívar, Caracas, Venezuela; ^25^Department of Cognitive Science and Psychology, New Bulgarian University, Sofia, Bulgaria; ^26^National Research Tomsk State University, Tomsk, Russia; ^27^Department of Biochemistry, Microbiology, and Immunology, University of Ottawa, Ottawa, ON, Canada; ^28^Oikostat GmbH, Ettiswil, Switzerland; ^29^School of Human Sciences, Senshu University, Kawasaki, Japan; ^30^Multimodal Interaction Lab, Leibniz-Institut für Wissensmedien, Tübingen, Germany; ^31^Consejo Nacional de Investigaciones Científicas y Técnicas, Mar del Plata, Argentina; ^32^Facultad de Psicología, Universidad Nacional de Mar del Plata, Mar del Plata, Argentina; ^33^Pontificia Universidad Católica de Valparaíso, Valparaíso, Chile; ^34^Vicerrectoría de Investigación y Desarrollo, Universidad Tecnológica de Chile INACAP, Santiago, Chile; ^35^Department of Medical and Surgical Sciences, “Magna Graecia” University of Catanzaro, Catanzaro, Italy; ^36^Economics Department, University of Campania “Luigi Vanvitelli”, Capua, Italy; ^37^Department of Psychosomatic Medicine, University Hospital Basel and University of Basel, Basel, Switzerland; ^38^Division of Clinical Psychology and Cognitive Behavioral Therapy, International Psychoanalytic University, Berlin, Germany; ^39^Division of Clinical Psychology and Epidemiology, Department of Psychology, University of Basel, Basel, Switzerland; ^40^Université Grenoble Alpes, Centre National de la Recherche Scientifique, LPNC, Grenoble, France; ^41^Department of Experimental Clinical and Health Psychology, Ghent University, Ghent, Belgium; ^42^Department of Mathematical Sciences, New Mexico State University, Las Cruces, NM, United States; ^43^Computational Statistics Laboratory (CAST), Department of Statistics, Universidade Federal de Pernambuco, Recife, Brazil; ^44^Business School, Massey University, Albany, New Zealand; ^45^Department of Psychology III, University of Würzburg, Würzburg, Germany; ^46^School of Psychology, National Autonomous University of Mexico, Mexico City, Mexico; ^47^CONSTRUCT-LESE, Faculty of Engineering, University of Porto, Porto, Portugal; ^48^FOM Hochschule für Oekonomie und Management, Essen, Germany; ^49^LEAD Graduate School & Research Network, University of Tübingen, Tübingen, Germany; ^50^Department of Psychology, Universidad del Norte, Barranquilla, Colombia; ^51^Division of Clinical Psychology and Psychiatry, Department of Psychology, University of Basel, Basel, Switzerland; ^52^Facultad de Ciencias Naturales y Exactas, Universidad de Playa Ancha, Valparaíso, Chile; ^53^Department of Methods and Statistics, Faculty of Social and Behavioural Sciences, Utrecht University, Utrecht, Netherlands; ^54^North-West University, Optentia Research Focus Area, Vanderbijlpark, South Africa; ^55^MINES Paristech, PSL Research University, Centre for Mathematical Morphology, Paris, France; ^56^Department of Mathematical Sciences, New Mexico State University, Las Cruces, NM, United States; ^57^Faculty of Arts and Science, Kyushu University, Fukuoka, Japan; ^58^School of Psychology, The University of Adelaide, Adelaide, SA, Australia

**Keywords:** statistical significance, null hypothesis testing, *p*-value, significance testing, decision making

## Abstract

We argue that making accept/reject decisions on scientific hypotheses, including a recent call for changing the canonical alpha level from *p* = 0.05 to *p* = 0.005, is deleterious for the finding of new discoveries and the progress of science. Given that blanket and variable alpha levels both are problematic, it is sensible to dispense with significance testing altogether. There are alternatives that address study design and sample size much more directly than significance testing does; but none of the statistical tools should be taken as the new magic method giving clear-cut mechanical answers. Inference should not be based on single studies at all, but on cumulative evidence from multiple independent studies. When evaluating the strength of the evidence, we should consider, for example, auxiliary assumptions, the strength of the experimental design, and implications for applications. To boil all this down to a binary decision based on a *p*-value threshold of 0.05, 0.01, 0.005, or anything else, is not acceptable.

Many researchers have criticized null hypothesis significance testing, though many have defended it too (see Balluerka et al., 2005, for a review). Sometimes, it is recommended that the alpha level be reduced to a more conservative value, to lower the Type I error rate. For example, Melton (1962), the editor of *Journal of Experimental Social Psychology* from 1950–1962, favored an alpha level of 0.01 over the typical 0.05 alpha level. More recently, Benjamin et al. (2018) recommended shifting to 0.005—consistent with Melton's comment that even the 0.01 level might not be “sufficiently impressive” to warrant publication (p. 554). In addition, Benjamin et al. (2018) stipulated that the 0.005 alpha level should be for new findings but were vague about what to do with findings that are not new. Though not necessarily endorsing significance testing as the preferred inferential statistical procedure (many of the authors apparently favor Bayesian procedures), Benjamin et al. (2018) did argue that using a 0.005 cutoff would fix much of what is wrong with significance testing. Unfortunately, as we will demonstrate, the problems with significance tests cannot be importantly mitigated merely by having a more conservative rejection criterion, and some problems are exacerbated by adopting a more conservative criterion.

We commence with some claims on the part of Benjamin et al. (2018). For example, they wrote “…changing the *P* value threshold is simple, aligns with the training undertaken by many researchers, and might quickly achieve broad acceptance.” If significance testing—at any *p*-value threshold—is as badly flawed as we will maintain it is (see also Amrhein et al., 2017; Greenland, 2017), these reasons are clearly insufficient to justify merely changing the cutoff. Consider another claim: “The new significance threshold will help researchers and readers to understand and communicate evidence more accurately.” But if researchers have understanding and communication problems with a 0.05 threshold, it is unclear how using a 0.005 threshold will eliminate these problems. And consider yet another claim: “Authors and readers can themselves take the initiative by describing and interpreting results more appropriately in light of the new proposed definition of statistical significance.” Again, it is not clear how adopting a 0.005 threshold will allow authors and readers to take the initiative with respect to better data interpretation. Thus, even prior to a discussion of our main arguments, there is reason for the reader to be suspicious of hasty claims with no empirical support.

With the foregoing out of the way, consider that a basic problem with tests of significance is that the goal is to reject a null hypothesis. This goal seems to demand—if one is a Bayesian—that the posterior probability of the null hypothesis should be low given the obtained finding. But the *p*-value one obtains is the probability of the finding, and of more extreme findings, given that the null hypothesis and all other assumptions about the model were correct (Greenland et al., 2016; Greenland, 2017), and one would need to make an invalid inverse inference to draw a conclusion about the probability of the null hypothesis given the finding. And if one is a frequentist, there is no way to traverse the logical gap from the probability of the finding and of more extreme findings, given the null hypothesis, to a decision about whether one should accept or reject the null hypothesis (Briggs, 2016; Trafimow, 2017). We accept that, by frequentist logic, the probability of a Type I error really is lower if we use a 0.005 cutoff for *p* than a 0.05 cutoff, all else being equal. We also accept the Bayesian argument by Benjamin et al. (2018) that the null hypothesis is less likely if *p* = 0.005 than if *p* = 0.05, all else being equal. Finally, we acknowledge that Benjamin et al. (2018) provided a service for science by further stimulating debate about significance testing. But there are important issues Benjamin et al. (2018) seem not to have considered, discussed in the following sections.

## Regression and replicability

Trafimow and Earp (2017) argued against the general notion of setting an alpha level to make decisions to reject or not reject null hypotheses, and the arguments retain their force even if the alpha level is reduced to 0.005. In some ways, the reduction worsens matters. One problem is that *p*-values have sampling variability, as do other statistics (Cumming, 2012). But the *p*-value is special in that it is designed to look like pure noise if the null hypothesis and all other model assumptions are correct, for in that case the *p*-value is uniformly distributed on [0,1] (Greenland, 2018). Under an alternative hypothesis, its distribution is shifted downwards, with the probability of *p* falling below the chosen cutoff being the power of the test. Because the actual power of typical studies is not very high, when the alternative is correct it will be largely a matter of luck whether the sampled *p*-value is below the chosen alpha level. When, as is often the case, the power is much below 50% (Smaldino and McElreath, 2016), the researcher is unlikely to re-sample a *p*-value below a significance threshold upon replication, as there may be many more *p*-values above than below the threshold in the *p*-value distribution (Goodman, 1992; Senn, 2002; Halsey et al., 2015). This problem gets worse as the cutoff is lowered, since for a constant sample size, the power drops with the cutoff.

Even if one did not use a cutoff, the phenomenon of regression to the mean suggests that the *p*-value obtained in a replication experiment is likely to regress to whatever the mean *p*-value would be if many replications were performed. How much regression should occur? When the null hypothesis is incorrect, that depends on how variable the point estimates and thus the *p*-values are.

Furthermore, the variability of *p*-values results in poor correlation across replications. Based on data placed online by the Open Science Collaboration (2015; https://osf.io/fgjvw), Trafimow and de Boer (submitted) calculated a correlation of only 0.004 between *p*-values obtained in the original cohort of studies with *p*-values obtained in the replication cohort, as compared to the expected correlation of zero if all the null hypotheses and models used to compute the *p*-values were correct (and thus all the *p*-values were uniformly distributed).

There are several possible reasons for the low correlation, including that most of the studied associations may have in fact been nearly null, so that the *p*-values remained primarily a function of noise and thus a near-zero correlation should be expected. But even if many or most of the associations were far from null, thus shifting the *p*-values downward toward zero and creating a positive correlation on replication, that correlation will remain low due not only to the large random error in *p*-values, but also due to imperfect replication methodology and the nonlinear relation between *p*-values and effect sizes (“correcting” the correlation for attenuation due to restriction of range, in the original cohort of studies, increases the correlation to 0.01, which is still low). Also, if most of the tested null hypotheses were false, the low *p*-value replicability as evidenced by the Open Science Collaboration could be attributed, in part, to the publication bias caused by having a publishing criterion based on *p*-values (Locascio, 2017a; Amrhein and Greenland, 2018). But if one wishes to make such an attribution, although it may provide a justification for using *p*-values in a hypothetical scientific universe where *p*-values from false nulls are more replicable because of a lack of publication bias, the attribution provides yet another important reason to avoid any sort of publishing criteria based on *p*-values or other statistical results (Amrhein and Greenland, 2018).

Thus, the obtained *p*-value in an original study has little to do with the *p*-value obtained in a replication experiment (which is just what the actual theory of *p*-values says should be the case). The best prediction would be a *p*-value for the replication experiment being vastly closer to the mean of the *p*-value distribution than to the *p*-value obtained in the original experiment. Under any hypothesis, the lower the *p*-value published in the original experiment (e.g., 0.001 rather than 0.01), the more likely it represents a greater distance of the *p*-value from the *p*-value mean, implying increased regression to the mean.

All this means that binary decisions, based on *p*-values, about rejection or acceptance of hypotheses, about the strength of the evidence (Fisher, 1925, 1973), or about the severity of the test (Mayo, 1996), will be unreliable decisions. This could be argued to be a good reason not to use *p*-values at all, or at least not to use them for making decisions on whether or not to judge scientific hypotheses as being correct (Amrhein et al., 2018).

## Error rates and variable alpha levels

Another disadvantage of using any set alpha level for publication is that the relative importance of Type I and Type II errors might differ across studies within or between areas and researchers (Trafimow and Earp, 2017). Setting a blanket level of either 0.05 or 0.005, or anything else, forces researchers to pretend that the relative importance of Type I and Type II errors is constant. Benjamin et al. (2018) try to justify their recommendation to reduce to the 0.005 level by pointing out a few areas of science which use very low alpha levels, but this observation is just as consistent with the idea that a blanket level across science is undesirable. And there are good reasons why variation across fields and topics is to be expected: A wide variety of factors can influence the relative importance of Type I and Type II errors, thereby rendering any blanket recommendation undesirable. These factors may include the clarity of the theory, auxiliary assumptions, practical or applied concerns, or experimental rigor. Indeed, Miller and Ulrich (2016) showed how these and other factors have a direct bearing on the final research payoff. There is an impressive literature attesting to the difficulties in setting a blanket level recommendation (e.g., Buhl-Mortensen, 1996; Lemons et al., 1997; Lemons and Victor, 2008; Lieberman and Cunningham, 2009; Myhr, 2010; Rice and Trafimow, 2010; Mudge et al., 2012; Lakens et al., 2018).

However, we do not argue that every researcher should get to set her own alpha level for each study, as recommended by Neyman and Pearson (1933) and Lakens et al. (2018), because that has problems too (Trafimow and Earp, 2017). For example, with variable thresholds, many old problems with significance testing remain unsolved, such as the problems of regression to the mean of *p*-values, inflation of effect sizes (the “winner's curse,” see below), selective reporting and publication bias, and the general disadvantage of forcing decisions too quickly rather than considering cumulative evidence across experiments. In view of all the uncertainty surrounding statistical inference (Greenland, 2017, 2018; Amrhein et al., 2018), we strongly doubt that we could successfully “control” error rates if only we would justify our alpha level and other decisions in advance of a study, as Lakens et al. (2018) seem to suggest in their comment to Benjamin et al. (2018). Nonetheless, Lakens et al. (2018) conclude that “the term ‘statistically significant’ should no longer be used.” We agree, but we think that significance testing with a justified alpha is still significance testing, whether the term “significance” is used or not.

Given that blanket and variable alpha levels both are problematic, it is sensible not to redefine statistical significance, but to dispense with significance testing altogether, as suggested by McShane et al. (2018) and Amrhein and Greenland (2018), two other comments to Benjamin et al. (2018).

## Defining replicability

Yet another disadvantage pertains to what Benjamin et al. (2018) touted as the main advantage of their proposal, that published findings will be more replicable using the 0.005 than the 0.05 alpha level. This depends on what is meant by “replicate” (see Lykken, 1968, for some definitions). If one insists on the same alpha level for the original study and the replication study, then we see no reason to believe that there will be more successful replications using the 0.005 level than using the 0.05 level. In fact, the statistical regression argument made earlier suggests that the regression issue is made even worse using 0.005 than using 0.05. Alternatively, as Benjamin et al. (2018) seem to suggest, one could use 0.005 for the original study and 0.05 for the replication study. In this case, we agree that the combination of 0.005 and 0.05 will create fewer unsuccessful replications than the combination of 0.05 and 0.05 for the initial and replication studies, respectively. However, this comes at a high price in arbitrariness. Suppose that two studies come in at *p* < 0.005 and *p* < 0.05, respectively. This would count as a successful replication. In contrast, suppose that the two studies come in at *p* < 0.05 and *p* < 0.005, respectively. Only the second study would count, and the combination would not qualify as indicating a successful replication. Insisting that setting a cutoff of 0.005 renders research more replicable would demand much more specificity with respect to how to conceptualize replicability.

In addition, we do not see a single replication success or failure as definitive. If one wishes to make a strong case for replication success or failure, multiple replication attempts are desirable. As is attested to by recent successful replication studies in cognitive psychology (Zwaan et al., 2017) and social sciences (Mullinix et al., 2015), the quality of the theory and the degree to which model assumptions are met will importantly influence replicability.

## Questioning the assumptions

The discussion thus far is under the pretense that the assumptions underlying the interpretation of *p*-values are true. But how likely is this? Berk and Freedman (2003) have made a strong case that the assumptions of random and independent sampling from a population are rarely true. The problems are particularly salient in the clinical sciences, where the falsity of the assumptions, as well as the divergences between statistical and clinical significance, are particularly obvious and dramatic (Bhardwaj et al., 2004; Ferrill et al., 2010; Fethney, 2010; Page, 2014). However, statistical tests not only test hypotheses but countless assumptions and the entire environment in which research takes place (Greenland, 2017, 2018; Amrhein et al., 2018). The problem of likely false assumptions, in combination with the other problems already discussed, render the illusory garnering of truth from *p*-values, or from any other statistical method, yet more dramatic.

## The population effect size

Let us continue with the significance and replication issues, reverting to the pretense that model assumptions are correct, while keeping in mind that this is unlikely. Consider that as matters now stand using tests of significance with the 0.05 criterion, the population effect size plays an important role both in obtaining statistical significance (all else being equal, the sample effect size will be larger if the population effect size is larger) and in obtaining statistical significance twice for a successful replication. Switching to the 0.005 cutoff would not lessen the importance of the population effect size, and would increase its importance unless sample sizes increased substantially from those currently used. And there is good reason to reject that replicability should depend on the population effect size. To see this quickly, consider one of the most important science experiments of all time, by Michelson and Morley (1887). They used their interferometer to test whether the universe is filled with a luminiferous ether that allows light to travel to Earth from the stars. Their sample effect size was very small, and physicists accept that the population effect size is zero because there is no luminiferous ether. Using traditional tests of significance with either a 0.05 or 0.005 cutoff, replicating Michelson and Morley would be problematic (see Sawilowsky, 2003, for a discussion of this experiment in the context of hypothesis testing). And yet physicists consider the experiment to be highly replicable (see also Meehl, 1967). Any proposal that features *p*-value rejection criteria forces the replication probability to be impacted by the population effect size, and so must be rejected if we accept the notion that replicability should not depend on population effect size.

In addition, with an alpha level of 0.005, large effect sizes would be more important for publication, and researchers might lean much more toward “obvious” research than toward testing creative ideas where there is more of a risk of small effects and of *p*-values that fail to meet the 0.005 bar. Very likely, a reason null results are so difficult to publish in sciences such as psychology is because the tradition of using *p*-value cutoffs is so ingrained. It would be beneficial to terminate this tradition.

## Accuracy of published effect sizes

It is desirable that published facts in scientific literatures accurately reflect reality. Consider again the regression issue. The more stringent the criterion level for publishing, the more distance there is from a finding that passes the criterion to the mean, and so there is an increasing regression effect. Even at the 0.05 alpha level, researchers have long recognized that published effect sizes likely do not reflect reality, or at least not the reality that would be seen if there were many replications of each experiment and all were published (see Briggs, 2016; Grice, 2017; Hyman, 2017; Kline, 2017; Locascio, 2017a,b; Marks, 2017 for a recent discussion of this problem). Under reasonable sample sizes and reasonable population effect sizes, it is the abnormally large sample effect sizes that result in *p*-values that meet the 0.05 level, or the 0.005 level, or any other alpha level, as is obvious from the standpoint of statistical regression. And with typically low sample sizes, statistically significant effects often are overestimates of population effect sizes, which is called “effect size inflation,” “truth inflation,” or “winner's curse” (Amrhein et al., 2017). Effect size overestimation was empirically demonstrated in the Open Science Collaboration (2015), where the average effect size in the replication cohort of studies was dramatically reduced from the average effect size in the original cohort (from 0.403 to 0.197). Changing to a more stringent 0.005 cutoff would result in yet worse effect size overestimation (Button et al., 2013; Amrhein and Greenland, 2018). The importance of having published effect sizes accurately reflect population effect sizes contradicts the use of threshold criteria and of significance tests, at any alpha level.

## Sample size and alternatives to significance testing

We stress that replication depends largely on sample size, but there are factors that interfere with researchers using the large sample sizes necessary for good sampling precision and replicability. In addition to the obvious costs of obtaining large sample sizes, there may be an underappreciation of how much sample size matters (Vankov et al., 2014), of the importance of incentives to favor novelty over replicability (Nosek et al., 2012) and of a prevalent misconception that the complement of *p*-values measures replicability (Cohen, 1994; Thompson, 1996; Greenland et al., 2016). A focus on sample size suggests an alternative to significance testing. Trafimow (2017; Trafimow and MacDonald, 2017) suggested a procedure as follows: The researcher specifies how close she wishes the sample statistics to be to their corresponding population parameters, and the desired probability of being that close. Trafimow's equations can be used to obtain the necessary sample size to meet this closeness specification. The researcher then obtains the necessary sample size, computes the descriptive statistics, and takes them as accurate estimates of population parameters (provisionally on new data, of course; an optimal way to obtain reliable estimation is via robust methods, see Huber, 1972; Tukey, 1979; Rousseeuw, 1991; Portnoy and He, 2000; Erceg-Hurn et al., 2013; Field and Wilcox, 2017). Similar methods have long existed in which sample size is based on the desired maximum width for confidence intervals.

This closeness procedure stresses (a) deciding what it takes to believe that the sample statistics are good estimates of the population parameters before data collection rather than afterwards, and (b) obtaining a large enough sample size to be confident that the obtained sample statistics really are within specified distances of corresponding population parameters. The procedure also does not promote publication bias because there is no cutoff for publication decisions. And the closeness procedure is not the same as traditional power analysis: First, the goal of traditional power analysis is to find the sample size needed to have a good chance of obtaining a statistically significant *p*-value. Second, traditional power analysis is strongly influenced by the expected effect size, whereas the closeness procedure is uninfluenced by the expected effect size under normal (Gaussian) models.

The larger point is that there are creative alternatives to significance testing that confront the sample size issue much more directly than significance testing does. The “statistical toolbox” (Gigerenzer and Marewski, 2015) further includes, for example, confidence intervals (which should rather be renamed and be used as “compatibility intervals”—see Amrhein et al., 2018; Greenland, 2018), equivalence tests, *p*-values as continuous measures of refutational evidence against a model (Greenland, 2018), likelihood ratios, Bayesian methods, or information criteria. And in manufacturing or quality control situations, also Neyman-Pearson decisions can make sense (Bradley and Brand, 2016).

But for scientific exploration, none of those tools should become the new magic method giving clear-cut mechanical answers (Cohen, 1994), because every selection criterion will ignore uncertainty in favor of binary decision making and thus produce the same problems as those caused by significance testing. Using a threshold for the Bayes factor, for example, will result in a similar dilemma as with a threshold for the *p*-value: as Konijn et al. (2015) suggested, “God would love a Bayes factor of 3.01 nearly as much as a Bayes factor of 2.99.”

Finally, inference should not be based on single studies at all (Neyman and Pearson, 1933; Fisher, 1937; Greenland, 2017), nor on replications from the same lab, but on cumulative evidence from multiple independent studies. It is desirable to obtain precise estimates in those studies, but a more important goal is to eliminate publication bias by including wide confidence intervals and small effects in the literature, without which the cumulative evidence will be distorted (Amrhein et al., 2017, 2018; Amrhein and Greenland, 2018). Along these lines, Briggs (2016) argues for abandoning parameter-based inference and adopting purely predictive, and therefore verifiable, probability models, and Greenland (2017) sees “a dire need to get away from inferential statistics and hew more closely to descriptions of study procedures, data collection […], and the resulting data.”

## Conclusion

It seems appropriate to conclude with the basic issue that has been with us from the beginning. Should *p*-values and *p*-value thresholds, or any other statistical tool, be used as the main criterion for making publication decisions, or decisions on accepting or rejecting hypotheses? The mere fact that researchers are concerned with replication, however it is conceptualized, indicates an appreciation that single studies are rarely definitive and rarely justify a final decision. When evaluating the strength of the evidence, sophisticated researchers consider, in an admittedly subjective way, theoretical considerations such as scope, explanatory breadth, and predictive power; the worth of the auxiliary assumptions connecting nonobservational terms in theories to observational terms in empirical hypotheses; the strength of the experimental design; and implications for applications. To boil all this down to a binary decision based on a *p*-value threshold of 0.05, 0.01, 0.005, or anything else, is not acceptable.

## Author contributions

All authors listed have made a direct contribution to the paper or endorse its content, and approved it for publication.

### Conflict of interest statement

FK-N was employed by Oikostat GmbH. GM has been acting as consultant for Janssen Research and Development, LLC. The other authors declare that the research was conducted in the absence of any commercial or financial relationships that could be construed as a potential conflict of interest.

## References

[B1] Amrhein V. Greenland S. (2018). Remove, rather than redefine, statistical significance. Nat. Hum. Behav. 2:4 10.1038/s41562-017-0224-0 30980046

[B2] Amrhein V. Korner-Nievergelt F. Roth T. (2017). The earth is flat (*p* > 0.05): significance thresholds and the crisis of unreplicable research. PeerJ. 5:e3544. 10.7717/peerj.3544 28698825 PMC5502092

[B3] Amrhein V. Trafimow D. Greenland S. (2018). Abandon statistical inference. PeerJ Preprints 6:e26857v1. 10.7287/peerj.preprints.26857v1

[B4] Balluerka N. Gómez J. Hidalgo D. (2005). The controversy over null hypothesis significance testing revisited. Methodology 1, 55–77. 10.1027/1614-1881.1.2.55

[B5] Benjamin D. J. Berger J. O. Johannesson M. Nosek B. A. Wagenmakers E.-J. Berk R. (2018). Redefine statistical significance. Nat. Hum. Behav. 2, 6–10. 10.1038/s41562-017-0189-z 30980045

[B6] Berk R. A. Freedman D. A. (2003). Statistical assumptions as empirical commitments, in Law, Punishment, and Social Control: Essays in Honor of Sheldon Messinger, 2nd Edn., eds Blomberg T. G. Cohen S. (New York, NY: Aldine de Gruyter), 235–254.

[B7] Bhardwaj S. S. Camacho F. Derrow A. Fleischer A. B. Feldman S. R. (2004). Statistical significance and clinical relevance. Arch. Dermatol. 140, 1520–1523. 10.1001/archderm.140.12.1520 15611433

[B8] Bradley M. T. Brand A. (2016). Significance testing needs a taxonomy: or how the Fisher, Neyman-Pearson controversy resulted in the inferential tail wagging the measurement dog. Psychol. Rep. 119, 487–504. 10.1177/0033294116662659 27502529

[B9] Briggs W. M. (2016). Uncertainty: The Soul of Modeling, Probability and Statistics. New York, NY: Springer.

[B10] Buhl-Mortensen L. (1996). Type-II statistical errors in environmental science and the precautionary principle. Mar. Pollut. Bull. 32, 528–531. 10.1016/0025-326X(96)00036-7

[B11] Button K. S. Ioannidis J. P. Mokrysz C. Nosek B. A. Flint J. Robinson E. S.. (2013). Power failure: why small sample size undermines the reliability of neuroscience. Nat. Rev. Neurosci. 14:365376. 10.1038/nrn3475 23571845

[B12] Cohen J. (1994). The earth is round (*p* < 0.05). Am. Psychol. 49, 997–1003.

[B13] Cumming G. (2012). Understanding the New Statistics: Effect Sizes, Confidence Intervals, and Meta-Analysis. New York, NY: Routledge.

[B14] Erceg-Hurn D. M. Wilcox R. R. Keselman H. J. (2013). Robust statistical estimation, in The Oxford Handbook of Quantitative Methods, Vol. 1, ed Little T. (New York, NY: Oxford University Press), 388–406.

[B15] Ferrill M. J. Brown D. A. Kyle J. A. (2010). Clinical versus statistical significance: interpreting *P* values and confidence intervals related to measures of association to decision making. J. Pharm. Pract. 23, 344–351. 10.1177/0897190009358774 21507834

[B16] Fethney J. (2010). Statistical and clinical significance, and how to use confidence intervals to help interpret both. Austr. Crit. Care 23, 93–97. 10.1016/j.aucc.2010.03.001 20347326

[B17] Field A. P. Wilcox R. R. (2017). Robust statistical methods: a primer for clinical psychology and experimental psychopathology researchers. Behav. Res. Ther. 98, 19–38. 10.1016/j.brat.2017.05.013 28577757

[B18] Fisher R. A. (1925). Statistical Methods for Research Workers, 1st Edn. Edinburgh: Oliver and Boyd.

[B19] Fisher R. A. (1937). The Design of Experiments, 2nd Edn. Edinburgh: Oliver and Boyd.

[B20] Fisher R. A. (1973). Statistical Methods and Scientific Inference, 3rd Edn. London: Macmillan.

[B21] Gigerenzer G. Marewski J. N. (2015). Surrogate science: the idol of a universal method for scientific inference. J. Manage. 41, 421–440. 10.1177/0149206314547522

[B22] Goodman S. N. (1992). A comment on replication, *p*-values and evidence. Stat. Med. 11, 875–879. 10.1002/sim.4780110705 1604067

[B23] Greenland S. (2017). The need for cognitive science in methodology. Am. J. Epidemiol. 186, 639–645. 10.1093/aje/kwx259 28938712

[B24] Greenland S. (2018). The unconditional information in *P*-values, and its refutational interpretation via *S*-values. Retrieved from: https://tinyurl.com/greenland2018

[B25] Greenland S. Senn S. J. Rothman K. J. Carlin J. B. Poole C. Goodman S. N.. (2016). Statistical tests, *P* values, confidence intervals, and power: a guide to misinterpretations. Eur. J. Epidemiol. 31, 337–350. 10.1007/s10654-016-0149-3 27209009 PMC4877414

[B26] Grice J. W. (2017). Comment on Locascio's results blind manuscript evaluation proposal. Basic Appl. Soc. Psych. 39, 254–255. 10.1080/01973533.2017.1352505

[B27] Halsey L. G. Curran-Everett D. Vowler S. L. Drummond G. B. (2015). The fickle *P* value generates irreproducible results. Nat. Methods 12, 179–185. 10.1038/nmeth.3288 25719825

[B28] Huber P. J. (1972). Robust statistics: a review. Ann. Math. Stat. 43, 1041–1067. 10.1214/aoms/1177692459

[B29] Hyman M. (2017). Can “results blind manuscript evaluation” assuage “publication bias”? Basic Appl. Soc. Psych. 39, 247–251. 10.1080/01973533.2017.1350581

[B30] Kline R. (2017). Comment on Locascio, results blind science publishing. Basic Appl. Soc. Psychol. 39, 256–257. 10.1080/01973533.2017.1355308 PMC5959282 29780193

[B31] Konijn E. A. van de Schoot R. Winter S. D. Ferguson C. J. (2015). Possible solution to publication bias through Bayesian statistics, including proper null hypothesis testing. Commun. Methods Meas. 9, 280–302. 10.1080/19312458.2015.1096332

[B32] Lakens D. Adolfi F. G. Albers C. J. Anvari F. Apps M. A. J. Argamon S. E. (2018). Justify your alpha. Nat. Hum. Behav. 2, 168–171. 10.1038/s41562-018-0311-x

[B33] Lemons J. Victor R. (2008). Uncertainty in river restoration, in River Restoration: Managing the Uncertainty in Restoring Physical Habitat, eds Darby S. Sear D. (Chichester: John Wiley and Sons), 3–13.

[B34] Lemons J. Shrader-Frechette K. Cranor C. (1997). The precautionary principle: scientific uncertainty and type I and type II errors. Found. Sci. 2, 207–236. 10.1023/A:1009611419680

[B35] Lieberman M. D. Cunningham W. A. (2009). Type I and Type II error concerns in fMRI research: re-balancing the scale. Soc. Cogn. Affect. Neurosci. 4, 423–428. 10.1093/scan/nsp052 20035017 PMC2799956

[B36] Locascio J. (2017a). Results blind science publishing. Basic Appl. Soc. Psychol. 39, 239–246. 10.1080/01973533.2017.1336093 PMC5959282 29780193

[B37] Locascio J. (2017b). Rejoinder to responses to “results-blind publishing.” Basic Appl. Soc. Psychol. 39, 258–261. 10.1080/01973533.2017.1356305 PMC6101654 30135613

[B38] Lykken D. T. (1968). Statistical significance in psychological research. Psychol. Bull. 70, 151–159. 10.1037/h0026141 5681305

[B39] Marks M. J. (2017). Commentary on Locascio 2017. Basic Appl. Soc. Psych. 39, 252–253. 10.1080/01973533.2017.1350580

[B40] Mayo D. (1996). Error and the Growth of Experimental Knowledge. Chicago, IL: The University of Chicago Press.

[B41] McShane B. B. Gal D. Gelman A. Robert C. Tackett J. L. (2018). Abandon statistical significance. arXiv:1709.07588v2.

[B42] Meehl P. E. (1967). Theory-testing in psychology and physics: a methodological paradox. Philos. Sci. 34, 103–115. 10.1086/288135

[B43] Melton A. (1962). Editorial. J. Exp. Psychol. 64, 553–557. 10.1037/h0045549

[B44] Michelson A. A. Morley E. W. (1887). On the relative motion of earth and luminiferous ether. Am. J. Sci. 34, 233–245. 10.2475/ajs.s3-34.203.333

[B45] Miller J. Ulrich R. (2016). Optimizing research payoff. Perspect. Psychol. Sci. 11, 664–691. 10.1177/1745691616649170 27694463

[B46] Mudge J. F. Baker L. F. Edge C. B. Houlahan J. E. (2012). Setting an optimal α that minimizes errors in null hypothesis significance tests. PLoS ONE 7:e32734. 10.1371/journal.pone.0032734 22389720 PMC3289673

[B47] Mullinix K. J. Leeper T. J. Druckman J. N. Freese J. (2015). The generalizability of survey experiments. J. Exp. Political Sci. 2, 109–138. 10.1017/XPS.2015.19

[B48] Myhr A. I. (2010). A precautionary approach to genetically modified organisms: challenges and implications for policy and science. J. Agricult. Environ. Ethics 23, 501–525. 10.1007/s10806-010-9234-x

[B49] Neyman J. Pearson E. S. (1933). On the problem of the most efficient tests of statistical hypotheses. Philos. Trans. R. Soc. Lond. Ser. A 231, 289–337. 10.1098/rsta.1933.0009

[B50] Nosek B. A. Spies J. R. Motyl M. (2012). Scientific utopia: II. Restructuring incentives and practices to promote truth over publishability. Perspect. Psychol. Sci. 7, 615–631. 10.1177/1745691612459058 26168121 PMC10540222

[B51] Open Science Collaboration (2015). Estimating the reproducibility of psychological science. Science 349:aac4716 10.1126/science.aac4716 26315443

[B52] Page P. (2014). Beyond statistical significance: Clinical interpretation of rehabilitation research literature. Int. J. Sports Phys. Ther. 9:72. 25328834 PMC4197528

[B53] Portnoy S. He X. (2000). A robust journey in the new millennium. J. Am. Stat. Assoc. 95, 1331–1335. 10.1080/01621459.2000.10474342

[B54] Rice S. Trafimow D. (2010). How many people have to die for a type II error? Theor. Issues Ergon. Sci. 11, 387–401. 10.1080/14639220902853096

[B55] Rousseeuw P. J. (1991). Tutorial to robust statistics. J. Chemom. 5, 1–20. 10.1002/cem.1180050103

[B56] Sawilowsky S. (2003). Deconstructing arguments from the case against hypothesis testing. J. Modern Appl. Stat. Methods 2, 467–474. 10.22237/jmasm/1067645940

[B57] Senn S. (2002). A comment on replication, *p*-values and evidence. Stat. Med. 21, 2437–2444. 10.1002/sim.1072 12210627

[B58] Smaldino P. E. McElreath R. (2016). The natural selection of bad science. R. Soc. Open Sci. 3:160384. 10.1098/rsos.160384 27703703 PMC5043322

[B59] Thompson B. (1996). AERA editorial policies regarding statistical significance testing: three suggested reforms. Educ. Res. 25, 26–30. 10.2307/1176337

[B60] Trafimow D. (2017). Using the coefficient of confidence to make the philosophical switch from *a posteriori* to *a priori* inferential statistics. Educ. Psychol. Meas. 77, 831–854. 10.1177/0013164416667977 PMC5965632 29795934

[B61] Trafimow D. Earp B. D. (2017). Null hypothesis significance testing and the use of P values to control the Type I error rate: the domain problem. New Ideas Psychol. 45, 19–27. 10.1016/j.newideapsych.2017.01.002

[B62] Trafimow D. MacDonald J. A. (2017). Performing inferential statistics prior to data collection. Educ. Psychol. Meas. 77, 204–219. 10.1177/0013164416659745 PMC5965545 29795910

[B63] Tukey J. W. (1979). Robust techniques for the user, in Robustness in Statistics, eds Launer R. L. Wilkinson G. N. (New York, NY: Academic Press), 103–106.

[B64] Vankov I. Bowers J. Munafò M. R. (2014). On the persistence of low power in psychological science. Q. J. Exp. Psychol. 67, 1037–1040. 10.1080/17470218.2014.885986 24528377 PMC4961230

[B65] Zwaan R. A. Pecher D. Paolacci G. Bouwmeester S. Verkoeijen P. Dijkstra K.. (2017). Participant Nonnaiveté and the reproducibility of cognitive psychology. Psychon. Bull. Rev. 10.3758/s13423-017-1348-y 28744765

